# Monitoring Water Sources for Environmental Reservoirs of Toxigenic *Vibrio cholerae* O1, Haiti

**DOI:** 10.3201/eid2003.131293

**Published:** 2014-03

**Authors:** Meer T. Alam, Thomas A. Weppelmann, Chad D. Weber, Judith A. Johnson, Mohammad H. Rashid, Catherine S. Birch, Babette A. Brumback, Valery E. Madsen Beau de Rochars, J. Glenn, Afsar Ali

**Affiliations:** University of Florida College of Public Health and Health Professions, Gainesville, Florida, USA (M.T. Alam, T.A. Weppelmann, V.E. Madsen Beau de Rochars, A. Ali);; University of Florida Emerging Pathogens Institute, Gainesville (M.T. Alam, T.A. Weppelmann, C.D. Weber, J.A. Johnson, M.H. Rashid, C.S. Birch, B.A. Brumback, V.E. Madsen Beau de Rochars, J.G. Morris, Jr., A. Ali);; University of Florida College of Medicine, Gainesville (M.H. Rashid, V.E. Madsen Beau de Rochars, A. Ali)

**Keywords:** *Vibrio cholerae*, O1, non-O1, 01, non-01, Haiti, environmental reservoirs, bacteria, waterborne, cholera, toxigenic

## Abstract

An epidemic of cholera infections was documented in Haiti for the first time in more than 100 years during October 2010. Cases have continued to occur, raising the question of whether the microorganism has established environmental reservoirs in Haiti. We monitored 14 environmental sites near the towns of Gressier and Leogane during April 2012–March 2013. Toxigenic *Vibrio cholerae* O1 El Tor biotype strains were isolated from 3 (1.7%) of 179 water samples; nontoxigenic O1 *V. cholerae* was isolated from an additional 3 samples. All samples containing *V. cholerae* O1 also contained non-O1 *V. cholerae*. *V. cholerae* O1 was isolated only when water temperatures were ≥31°C. Our data substantiate the presence of toxigenic *V. cholerae* O1 in the aquatic environment in Haiti. These isolations may reflect establishment of long-term environmental reservoirs in Haiti, which may complicate eradication of cholera from this coastal country.

Epidemic cholera was identified during October 2010 in Haiti; initial cases were concentrated along the Artibonite River (*1*,*2*). The clonal nature of isolates during this initial period of the epidemic has been described (*3*–*6*). Because cholera had not been reported in Haiti for at least 100 years, there is a high likelihood that the responsible toxigenic *Vibrio cholerae* strain was introduced into Haiti, possibly through Nepalese peacekeeping troops garrisoned at a camp along the Artibonite River (*4*,*7*). In the months after October 2010, cholera spread quickly through the rest of Haiti: 604,634 cases and 7,436 deaths were reported in the first year of the epidemic (*1*). In the intervening years, cases and epidemics have been reported, and it has been suggested that onset of the rainy season serves as a trigger for disease occurrences (*2*,*8*).

*V. cholerae* is well recognized as an autochthonous aquatic microorganism species with the ability to survive indefinitely in aquatic reservoirs and is possibly in a “persister” phenotype (*9*). *V. cholerae* strains can also persist in aquatic reservoirs as a rugose variant that promotes formation of a biofilm that confers resistance to chlorine and to oxidative and osmotic stresses (*10*–*13*) and also persists in a viable but nonculturable form (*14*). Work by our group and others suggests that cholera epidemics among humans are preceded by an environmental bloom of the microorganism and subsequent spillover into human populations (*15*–*17*). In our studies in Peru (*16*), water temperature was found to be the primary trigger for these environmental blooms and could be correlated with subsequent increases in environmental counts and occurrence of human illness.

To understand patterns of ongoing cholera transmission and seasonality of cholera in Haiti, and to assess the likelihood of future epidemics, it is essential to know whether environmental reservoirs of toxigenic *V. cholerae* O1 have been established, where these reservoirs are located, and what factors affect the occurrence and growth of the microorganism in the environment. We report the results of an initial year of monitoring of environmental sites in the Ouest Department of Haiti, near the towns of Leogane and Gressier, where the University of Florida (Gainesville, FL, USA) has established a research laboratory and field area.

## Methods

### Environmental Sampling Sites

**Figure 1:**
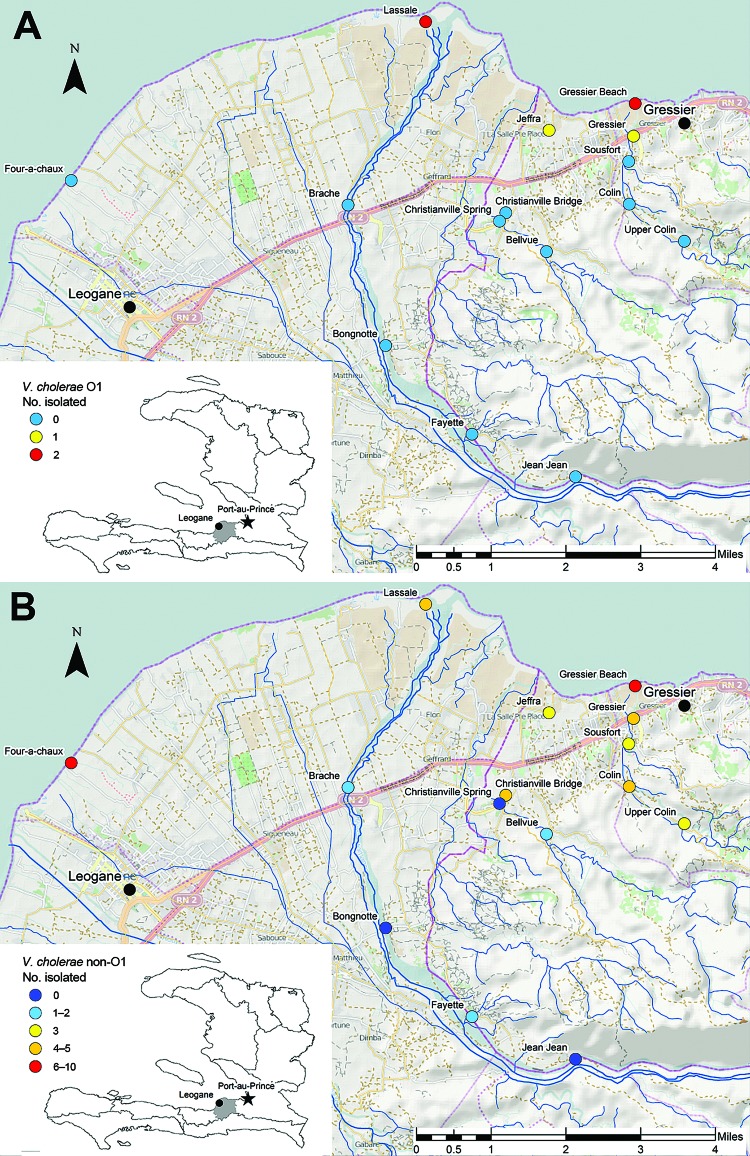
Locations of environmental sampling sites near the towns of Gressier and Leogane in Haiti. Samples were collected during April 2012–March 2013. A) Number of *Vibrio cholerae* O1 isolates obtained from sampling sites. B) Number of non-O1/non-O139 *V. cholerae* isolates obtained from sampling sites. The number of *V. cholerae* isolates obtained from each sampling site is indicated by distinct color coding.

**Figure 2:**
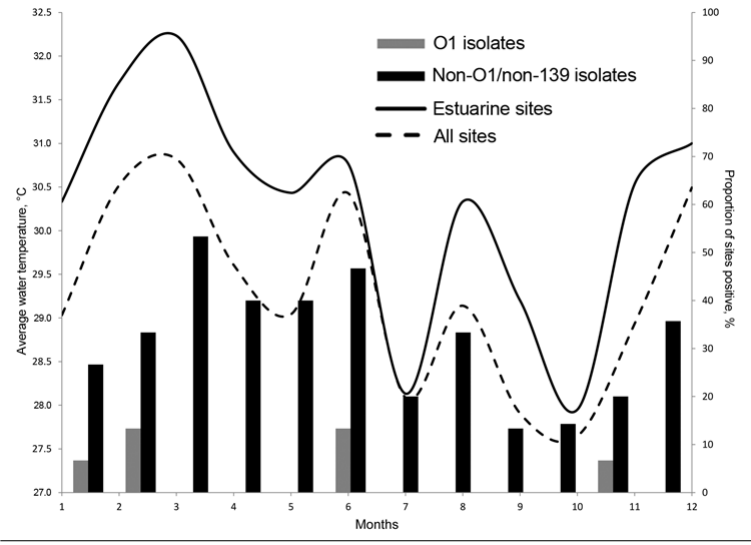
Mean combined water temperature for all sites monitored in the Ouest Department of Haiti, near the towns of Leogane and Gressier, and percentage of environmental sites positive for *Vibrio cholerae* O1 or non-O1/non-O139, by month.

Fifteen fixed environmental sampling sites were selected near Gressier and Leogane (Figures 1,2). Sites were selected along transects of 3 rivers in the area and at 1 independent estuarine site: the Momance River (4 up-river sites and 1 estuarine site at the mouth of the river), the Gressier River (4 up-river sites and1 estuarine site at the mouth of the river), the Tapion River (4 river sites), and an independent estuarine site at Four-a-chaux, which is a historic ruin and tourist attraction. All sites were >0.5 miles apart, with the exception of the Christianville Bridge and Spring sites, which were 0.25 miles apart. Topography of this area is typical for Haiti: rivers originated in the mountains (peaks in the region are >8,000 feet) and flowed into a broad flood plain where Gressier and Leogane were located. Up-river sites on the Momance and Gressier Rivers were in the mountains, where human populations are limited. Water samples were collected once a month from each site during April 2012–March 2013. A total of 179 samples were collected for culture for *V. cholerae*; 176 samples were available for measurement of water quality parameters.

### Isolation and Identification of *V. cholerae* from Environmental Sites

For the isolation of *V. cholerae,* 500 ml of water was collected in a sterile 500-mL Nalgene (http://nalgene.com/) bottle from each fixed site; the samples were transported at ambient temperature to the University of Florida laboratory at Gressier and processed for detection of *V. cholerae* within 3 hours of collection.

In addition to the conventional sample enrichment technique (*18*), we used alkaline peptone water (APW) to enrich water samples. A 1.5-mL water sample was enriched with 1.5 mL of 2× APW in 3 tubes: 1 tube was incubated at 37°C for 6–8 hours (*18*), another tube was incubated overnight at 37°C, and the third tube was incubated at 40°C for 6–8 hours. Subsequently, a loopful of culture from each tube was streaked onto thiosulfate citrate bile salts sucrose agar (Becton-Dickinson, Franklin Lakes, NJ, USA), and the plates were incubated overnight at 37°C. From each plate, 6–8 yellow colonies exhibiting diverse morphology were transferred to L-agar; these plates were incubated overnight at 37°C. Each colony was examined by using the oxidase test; oxidase-positive colonies were tested by using *V. cholerae* O1–specific polyvalent antiserum and O139-specific antiserum (DENKA SEIKEN Co., Ltd, Tokyo, Japan). The isolates were further examined by using colony PCR for the presence of *ompW* and *toxR* genes specific for *V. cholerae* spp. as described (*9*).

### Screening of Aquatic Animals and Plants

To determine whether they serve as reservoirs for *V. cholerae* O1, we collected aquatic animals typically eaten by humans, including shrimp, fish, crab, crayfish, and aquatic plants (n = 144) weekly during February 5–22, 2013. The samples were collected from 14 environmental sites. Each sample was placed into a sterile plastic seam–locking bag and transported to the laboratory. One gram of the sample was mixed with 100 mL of saline and then homogenized in a sterile blender; 1.5 ml of the resultant mixture was enriched in 2× APW and processed as described.

### Genetic Characterization of *V. cholerae* O1 Strains

To further characterize the environmental *V. cholerae* O1 serogroup Ogawa biotype El Tor strains, we subjected all *V. cholerae* O1 isolates from water and seafood to PCR analysis for key virulence genes, including *ctxA*, *ctxB^-CL^*, (MAMA^-CL^), *ctxB*-^ET^ (MAMA^-ET^), *rstR*^-ET^, *rstR*^-CL^, *rstC*^-ET^, *rstC*^-CL^, *tcpA*^-CL^, and *tcpA*^-ET^, as described (*19*,*20*). The chromosomal DNA was extracted from each strain by using a GenElute Bacterial Genomic DNA kit (Sigma-Aldrich, St. Louis, MO, USA), and the DNA was used for PCR templates; the PCR conditions were as described (*3*).

### Aerobic Plate Counts

To determine total aerobic bacterial counts in water samples, we plated undiluted, 10- and 100-fold dilutions of water onto L-agar and incubated overnight at 37°C. The countable plates (100–300 colonies) were used to determine the total (CFU/mL) culturable bacteria present in the water samples.

### Water Parameters, Rainfall, and Human Case Counts

When collecting water samples, we measured physical parameters, including pH, water temperature, dissolved oxygen, total dissolved solids, salinity, and conductivity in the field sites by using a HACH portable meter (HACH Company, Loveland, CO, USA) and designated electrodes following the manufacturer’s recommendations. Rainfall estimates were based on National Aeronautics and Space Administration data for the study region bounded by the rectangle (18.2°–18.6°N, 17.1°–17.8°W) by using the average daily rainfall measurement tool, Tropical Rainfall Measuring Mission 3B42_daily (*21*). Estimates of average precipitation (mm/day) with a spatial resolution of 0.25 × 0.25 degrees were aggregated to obtain weekly accumulated rainfall measurements during the study period. Cholera incidence data were obtained from daily reports by Ouest Department (excluding Port-au-Prince) to the Haitian Ministry of Public Health and Population and aggregated to total cases per week during April 20, 2012­–March 27, 2013 (*22*).

### Data Analysis 

We examined the effects of water quality factors on the presence of toxigenic and nontoxigenic *V. cholerae* by conditional logistic regression after stratification for the site. Stratification excluded sites that had all-positive or all-negative outcomes; of the remaining sites, regression analysis showed O1 *V. cholerae* in 47 observations from 4 sites and non-O1/non-O139 *V. cholerae* in 154 observations from13 sites. As shown in Figure 1, we performed cartography by using ArcGIS version 10 (ESRI, Redlands, CA, USA).

## Results

*V. cholerae* O1 serogroup Ogawa biotype El Tor was isolated from 6 (3.4%) of the 179 water samples and 1 (0.7%) of the 144 aquatic animal and plant samples by using modified APW enrichment techniques. Of those 7 environmental isolates, 3 (43%) were confirmed as *ctx*-positve toxigenic *V. cholerae* O1 strains, and 4 (57%) were confirmed as *ctx*-negative *V. cholerae* O1 strains by using genetic analysis as described below. As shown in Table 1, APW enrichment at 37°C overnight or incubation at 40°C for 6–8 hours, or both, enhanced the rate of isolation of *V. cholerae* O1 from samples. PCR analysis of the key virulence genes showed that 3 (43%) of the 7 isolates, all from water, were positive for key virulence genes, including cholera toxin genes and *tcpA* genes, and that 4 (57%) isolates exhibited no cholera toxin bacteriophage (CTXΦ)–related genes (*23*; Table 2). To further assess the PCR results, we sequenced DNA flanking the CTXΦ from 1 strain, Env-9 (Table 2). Sequence data corroborated PCR results that indicated that Env-9 lacked CTXΦ. 

**Table 1:** Effect of diverse enrichment conditions on the isolation of culturable *Vibrio cholerae* O1 strains from aquatic reservoirs in the Gressier and Leogane regions of Haiti

Strain ID	Culture results after alkaline peptone water enrichment		
	37°C (6–8 h)	37°C (18–24 h)	40°C (6–8 h)
Env-9	-	-	+
Env-90	-	-	+
Env-94	+	-	+
Env-122*	+	-	-
Env-383	-	+	-
Env-390	+	-	-
Env-114*	-	+	+

*Env-122 and env-114 were isolated from water and a shrimp sample, respectively, from a single sampling site at a single isolation round.

**Table 2:** PCR analysis of genes of *ctx* –positive toxigenic *Vibrio cholerae* O1 strains and *ctx*-negative *V. cholerae* O1 strain

Strain	PCR											Mismatch amplification mutation assay PCR	
	*ompW*	*toxR*	*tcpA* ^CL^	*tcpA* ^ET^	*ctxA*	*ctxB*	*rstR* ^ET^	*rstR* ^CL^	*rstC* ^ET^	*rstC* ^CL^		*ctxB* ^CL^	*ctxB* ^ET^
Env-9*	+	+	+	-	-	-	-	-	-	-		-	-
Env-90	+	+	-	+	+	+	+	-	-	-		+	-
Env-94	+	+	-	+	+	+	+	-	-	-		+	-
Env-122*†	+	+	+	-	-	-	-	-	-	-		-	-
Env-383	+	+	-	+	+	+	+	-	-	-		+	-
Env-390*	+	+	+	-	-	-	-	-	-	-		-	-
Env-114*†	+	+	+	-	-	-	-	-	-	-		-	-

*Indicates *ctx-*negative *V. cholerae* O1 isolates. †Env-122 and env-114 were isolated from water and a shrimp sample, respectively, from a single sampling site and at a single isolation round.

**Figure 3:**
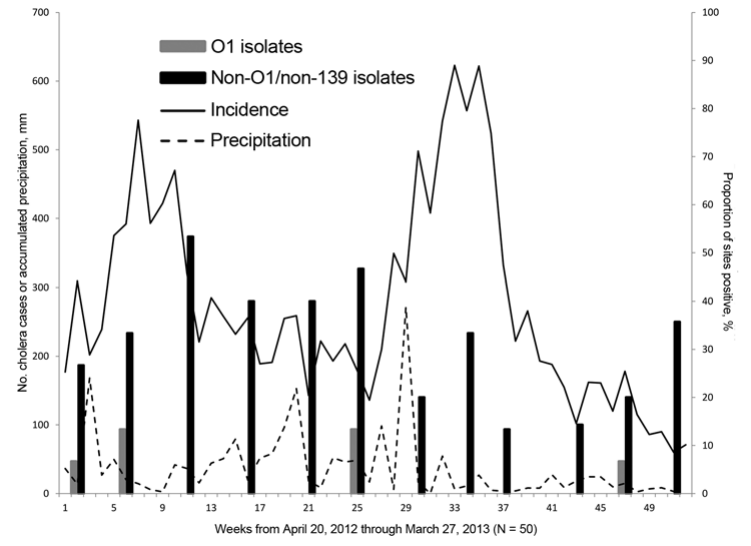
Weekly cholera case incidence for Ouest Department, excluding Port-au-Prince, Haiti, based on data reported to the Haitian Ministry of Public Health and Population and regional precipitation by week during April 2012–March 2013, combined with percentage of environmental sites from which *V. cholerae* O1 or non-O1/non-O139 were isolated, by month.

Physical parameters for the environmental water samples are summarized in Table 3. Because the sites varied from mountains to floodplain to estuaries, there was relatively wide variability in salinity (0–21.6 g/L), pH (6.4–8.6), and temperature (24.3–33.7°C). Temperatures tended to increase as rivers approached the sea. As shown in Figure 2, mean water temperature from all sites showed evidence of seasonal variation. Measurement of rainfall was available for the region as a whole (Figure 3). However, site-specific rainfall data were not available; consequently, rainfall was not included in the regression models.

**Table 3:** Summary statistics of environmental water quality factors in mountains, estuaries, and a floodplain in Haiti, April 2012–March 2013

Water quality	No. specimens observed	Mean	SD	Minimum	Maximum
Temperature, °C	176	29.32	1.91	24.3	33.7
pH, log[H+]	176	7.71	0.36	6.4	8.6
Dissolved oxygen, mg/L	176	7.33	1.81	1.23	10.31
Total dissolved solids, mg/L	176	273.8	359.9	24	2,970
Salinity, g/L	176	0.38	1.72	0	21.6
Conductivity (µS/cm)	176	544.1	639.4	230	5,630
Heterotrophic bacteria, log(cfu/mL)	175	4.21	0.60	2.3	5.89

Isolation of *V. cholerae* O1 strains was most common from the sites at the mouths of the Momance and Gressier Rivers (Figure 1, panel A). In a conditional logistic regression analysis with water quality factors (Table 4), the only variable that emerged as statistically significant was water temperature (odds ratio 2.14, 95% CI 1.06–4.31); all isolations of *V. cholerae* O1 (toxigenic and nontoxigenic) occurred at water temperatures of >31°C. As shown in Figure 3, there was evidence that *V. cholerae* O1 isolation was more common in the environment preceding epidemic peaks of disease among humans; however, numbers of isolations were too small to permit statistical analysis. Of 179 samples, the only *V. cholerae* O1 isolate from aquatic animals or plants was from a shrimp sample and was nontoxigenic; it was collected simultaneously with a water sample that was also positive for nontoxigenic *V. cholerae* O1.

**Table 4:** Conditional logistic regression analysis of water quality factors affecting the occurrence *Vibrio cholerae* O1 and non-O1/non-O139 in aquatic reservoirs, Haiti, April 2012–March 2013

Factor	Units	No. observations	Odds ratio (95% CI)	p value
Presence of *V. cholera* 01				
Temperature	1°C	47	2.14 (1.06–4.31)	0.033*
pH	1 log[H+]	47	0.01 (0.00–1.81)	0.083
Dissolved oxygen	1 mg/L	47	0.32 (0.08–1.20)	0.091
Total dissolved solids	100 mg/L	47	1.08 (0.95–1.23)	0.258
Salinity	1 g/L	47	1.24 (0.86–1.80)	0.254
Conductivity	100 (µS/cm)	47	1.05 (0.98–1.13)	0.198
Heterotrophic bacteria	log (CFU/mL)	47	6.00 (0.57–62.78)	0.135
Presence of *V. cholera* non-01				
Temperature	1°C	154	1.36 (1.05–1.76)	0.02*
pH	1 log[H+]	154	0.44 (0.09–2.14)	0.311
Dissolved oxygen	1 mg/L	154	0.50 (0.32–0.79)	0.003*
Total dissolved solids	100 mg/L	154	0.96 (0.86–1.06)	0.413
Salinity	1 g/L	154	1.19 (0.80–1.77)	0.378
Conductivity	100 (µS/cm)	154	0.98 (0.92–1.04)	0.432
Heterotrophic bacteria	log (CFU/mL)	153	2.35 (0.95–5.77)	0.063

*p<0.05 were considered statistically significant.

Non-O1 *V. cholerae* was much more common in the environment than *V. cholerae* O1 strains and was isolated from 56 (31%) of 179 water samples. As observed with O1 strains, isolations were more common at the mouths of the rivers and in estuarine areas (Figure 1, panel B); however, the non-O1 strain was found farther upriver than were O1 strains and was isolated from several sites in the mountains. Non-O1 strains were isolated from all sites that were also positive for O1 strains. Non-O1 strains were isolated in all months, without an obvious association with regional rainfall totals or cholera incidence. In a conditional logistic regression analysis, isolation of non-O1 strains was significantly associated (p<0.05) with higher water temperature and moderate levels of dissolved oxygen (Table 4).

## Discussion

Before this study, isolation of 2 toxigenic *V. cholerae* O1 strains from large-volume water samples (30 L) was reported in the Artibonite region (*24*); other studies at that time suggested that *V. cholerae* O1 strains were not present, or present at only minimal levels (*2*,*25*) in the environment in Haiti. In contrast, we isolated *ctx*-positive and *ctx*-negative *V. cholerae* O1 serogroup Ogawa biotype El Tor strains (Table 1) in the environment at a frequency comparable to that reported from cholera-endemic areas such as Bangladesh (*17*). Our successful isolation of the microorganism from the environment may reflect localization of environmental isolates near Gressier and Leogane, where our study was conducted; however, we believe that our findings are more likely to be a reflection of the method used. Data presented here suggest that, in addition to conventional APW enrichment, longer APW enrichment time and enrichment at higher temperatures contributed to an increased rate of isolation of *V. cholerae* O1 strains from aquatic environmental reservoirs (Table 1), resulting in successful isolation from 1.5-mL water samples. We also note some issues relating to sample transport: Baron et al. (*25*) transported their water samples on ice in coolers; our samples were transported at room temperature. As has been reported, *Vibrio* spp. are extremely sensitive to low temperatures (*26*), and in our experience, transport of samples on ice resulted in a marked reduction in isolation rates.

Water from which we isolated *V. cholerae* spp. tended to have been sampled at the point where rivers meet the sea, and in adjacent estuarine areas, again following the patterns reported from Bangladesh (*17*). Water temperature was found to be the single physical parameter that was substantially associated with isolation of these organisms; higher temperatures were concentrated downriver and in estuarine areas. For our analysis, we used a conditional logistic regression model to permit stratification by site. Although we found very low numbers for *V. cholerae* O1 isolates (6 positive water samples), results coincided with the non-O1 results and the exploratory data analysis. In our studies of aquatic animals likely to be eaten by humans, we did isolate *V. cholerae* O1 from shrimp in 1 instance. The isolate was nontoxigenic; consequently, its association with disease is unclear.

After analyzing the results of this study, we asked the following question: has *V. cholerae* O1 become established in environmental reservoirs in Haiti? Toxigenic *V. cholerae* O1 strains are clearly present in the environment, and it may be that the isolates that we identified are the result of fecal contamination of the environment by persons infected with *V. cholera* strains. Although data are limited, there was at least a suggestion that isolation of *V. cholera* strains from environmental reservoirs was more common at the beginning of epidemic spikes of human disease (as has been described in association with environmental reservoirs) (*16*,*17*,*27*) rather than at the height of epidemics among humans, as might have been expected related to fecal contamination. We also found non-O1 strains widely distributed throughout the environment, including mountain river sites, consistent with widespread dissemination in environmental reservoirs. Although we cannot be certain that O1 and non-O1 strains grow under comparable conditions, the clear establishment of non-O1 *V. cholerae* strains in environmental reservoirs suggests that conditions are appropriate for growth of *V. cholerae* O1 strains. Of potentially greater interest is the observation that only 3 of the 7 (47%) *V. cholerae* O1 biotype El Tor strains isolated carried the *ctx* genes (Table 2). Data from 1 *ctx*-negative strain (Env-9) was consistent with absence of the entire CTXΦ. We propose that the 3 isolates that are positive for *ctx* genes be classified as circulating *V. cholerae* altered biotype El Tor strains in Haiti. To better understand the evolutionary mechanisms involved, we are performing further sequence analysis of clinical and environmental strains. 

## Conclusions

The apparent introduction of toxigenic *V. cholerae* O1 in Haiti in 2010, after decades during which no cholera cases were reported, was unquestionably a public health disaster. If these O1 strains establish stable environmental reservoirs in Haiti, in the setting of ongoing problems with water and sanitation, there is a high likelihood that we will see recurrent epidemics within the country. These circumstances clearly have implications for current plans by the Haitian Ministry of Public Health to eradicate cholera in Haiti within a decade (*28*). The proposed implementation of vaccination programs and efforts to improve water supplies and sanitation will undoubtedly reduce case numbers, but as long as the causative microorganism is present in the environment, eradication of the disease will not be possible. Establishment of environmental reservoirs and recurrent epidemics may also serve as a potential source for transmission of the disease to the Dominican Republic and other parts of the Caribbean (*1*). Ongoing monitoring of potential environmental reservoirs in the areas near Gressier and Leogane as well as in sentinel sites throughout the country will be necessary to assess this risk and to permit development of rational public health interventions for cholera control.
